# Heteroatom-Coordinated Fe–N_4_ Catalysts for Enhanced Oxygen Reduction in Alkaline Seawater Zinc-Air Batteries

**DOI:** 10.1007/s40820-025-01943-6

**Published:** 2026-01-03

**Authors:** Wenhan Fang, Kailong Xu, Xinlei Wang, Yuanhang Zhu, Xiuting Li, Hui Liu, Danlei Li, Jun Wu

**Affiliations:** 1 Department of School of Metallurgy and Environment, Central South University, Changsha, 410083 People’s Republic of China; 2 State Key Laboratory of Advanced Metallurgy for Non-Ferrous Metals, Changsha, 410083 People’s Republic of China; 3 Department of Chemistry and Materials Science, School of Science, Xi’an Jiaotong-Liverpool University, Suzhou, 215123 People’s Republic of China; 4 Department of Chemistry, University of Liverpool, Liverpool, L69 7ZD UK; 5 Institute for Advanced Study, Shenzhen University, Shenzhen, 518060 People’s Republic of China

**Keywords:** Single-atom catalyst, Zinc-air battery, Seawater catalyst, Oxygen reduction reaction

## Abstract

Seawater zinc-air batteries are promising energy storage devices due to their high energy density and utilization of seawater electrolytes. However, their efficiency is hindered by the sluggish oxygen reduction reaction (ORR) and chloride-induced degradation over conventional catalysts. In this study, we proposed a universal synthetic strategy to construct heteroatom axially coordinated Fe–N_4_ single-atom seawater catalyst materials (Cl–Fe–N_4_ and S–Fe–N_4_). X-ray absorption spectroscopy confirmed their five-coordinated square pyramidal structure. Systematic evaluation of catalytic activities revealed that compared with S–Fe–N_4_, Cl–Fe–N_4_ exhibits smaller electrochemical active surface area and specific surface area, yet demonstrates higher limiting current density (5.8 mA cm^−2^). The assembled zinc-air batteries using Cl–Fe–N_4_ showed superior power density (187.7 mW cm^−2^ at 245.1 mA cm^−2^), indicating that Cl axial coordination more effectively enhances the intrinsic ORR activity. Moreover, Cl–Fe–N_4_ demonstrates stronger Cl^−^ poisoning resistance in seawater environments. Chronoamperometry tests and zinc-air battery cycling performance evaluations confirmed its enhanced stability. Density functional theory calculations revealed that the introduction of heteroatoms in the axial direction regulates the electron center of Fe single atom, leading to more active reaction intermediates and increased electron density of Fe single sites, thereby enhancing the reduction in adsorbed intermediates and hence the overall ORR catalytic activity.

**Supplementary Information:**

The online version contains supplementary material available at 10.1007/s40820-025-01943-6.

## Highlights

- A universal synthetic strategy was proposed to construct heteroatom axially coordinated Fe–N_4_ single-atom seawater catalyst materials (Cl–Fe–N_4_ and S–Fe–N_4_).
- The Cl–Fe–N_4_ catalyst achieves a limiting current density of 5.8 mA cm^−2^ and a half-wave potential of 0.931 V *vs.* RHE in alkaline synthetic seawater, outperforming commercial Pt/C (40 wt%).
- The seawater-based zinc-air battery fabricated with Cl–Fe–N_4_ demonstrates a power density of 187.7 mW cm^−2^ at 245.1 mA cm^−2^ and maintains stable cycling performance for 200 h.

**Supplementary Information:**

The online version contains supplementary material available at 10.1007/s40820-025-01943-6.

## Graphical abstract

**Figure:**
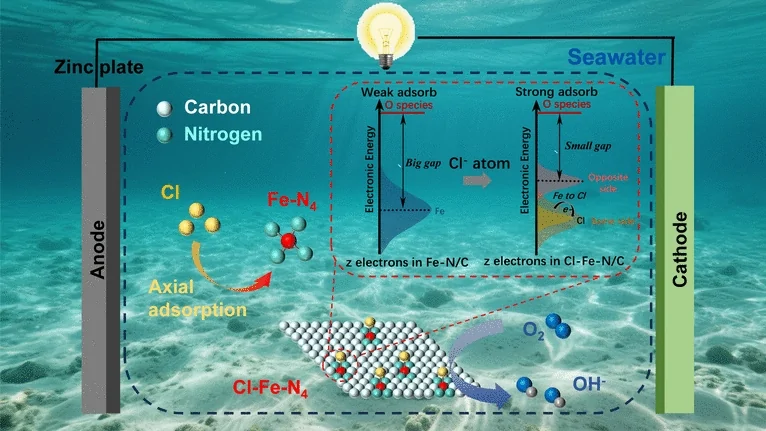

## Introduction

Seawater zinc-air batteries (SZABs) have emerged as a promising energy storage technology due to their high energy density, environmental sustainability, and direct utilization of abundant seawater as electrolyte [1–3]. Unlike conventional batteries, SZABs uses seawater as electrolyte due to its high ionic conductivity of seawater rather, avoiding the need of fresh water resources and enabling cost-effective and scalable applications in marine [4–6]. One of the key issues which limit the overall efficiency and energy output of SZAB is the sluggish oxygen reduction reaction (ORR) [7–9]. The inherently slow kinetics due to the 4-electron transfer process requires high-performance electrocatalysts to accelerate reaction rates and reduce overpotentials [10]. Although noble metal-based electrocatalysts such as Pt exhibit excellent catalytic performance [11–14], their high costs limit the practical applications in energy-related devices [15, 16]. Moreover, another major challenge to metal-based catalyst in seawater environment is the poor stability due to the presence of high concentration of Cl^−^, which can easily adsorb onto metal center, resulting in a decrease in catalytic active site and even shifting the ORR pathway from the preferred four-electron reduction of O_2_ to H_2_O toward an unfavorable two-electron reduction to H_2_O_2_ [17–19]. Additionally, Cl^−^-induced corrosion and the formation of inactive metal-chloride (M-Cl) species further exacerbate performance degradation [20]. To address these challenges, extensive research has been focused on the development of electrocatalysts which not only enhance ORR activity but also exhibit strong resistance to Cl^−^ poisoning and side reactions.

Single-atom catalysts (SACs) have attracted significant attention due to their maximized atomic utilization efficiency, well-defined active sites, and tunable electronic structures [21]. Their atomically dispersed metal centers also minimize aggregation and enhance stability under various electrochemical conditions [22]. Recent studies have demonstrated that Fe-based SACs showed comparable performance as Pt-based catalysts in alkaline ORR, sometime even better, particularly when their coordination environments are optimized [23–25]. Current strategies focus on modifying the electronic structure of active sites or engineering protective layer around active sites to mitigate undesired interactions with seawater components. The former can improve the adsorption capacity of active sites for O_2_ by adjusting electron delocalization [26], and the relevant literature has revealed a potential-dependent dynamic evolution of active sites: At high potentials (≥ 0.4 V), Fe–(H_2_O)N_4_ dominates; at medium to low potentials, O_2_-assisted FeN_4_ or Fe–(OH)N_4_ predominates. Oxygen ligands promote *OH protonation by regulating the electron occupancy of Fe 3dz^2^↑/3dxz↓ orbitals [27]. Therefore, modulating the electronic structure of Fe–N_4_ sites is central to enhancing ORR performance [28, 29]. Examples include regulating the magnetic moment of Fe through the introduction of adjacent copper atoms [30], and the axial Fe–O coordination formed by FePc with an oxygen-containing carbon substrate (AB–O), which breaks the planar symmetry of FeN_4_, inducing electron localization on the axial O atom to achieve ORR performance enhancement [31]. Another example is the introduction of external nitrogen (such as pyrrolic nitrogen, PN) to modulate the charge distribution of FeN_4_ sites, thereby enhancing the positive charge of Fe and local electric field distortion, significantly boosting ORR activity [32]. The latter is usually achieved by so-called carbon layer functionalization to enhance the Cl^−^ corrosion resistance of catalysts in seawater environments [33]. Recent research has revealed that by introducing Cl^−^ adsorption directly at metal sites such as Fe or Ag can enhance local Cl^−^ concentration, and the charge environment can therefore be modified to improve the oxygen affinity and Cl^−^-selective repulsion of metal sites [22, 34]. While Cl^−^ inhibition strategies exist, the impact of different heteroatom dopants on Fe-based SACs in seawater remains unclear.

The synthesis methods for currently reported heteroatom-doped catalysts encompass various approaches. The most common method is pyrolysis, typically involving the thermal decomposition of mixtures containing heteroatom precursors and carbon sources at temperatures ranging from 600 to 1000 °C to yield catalysts [35]. Additionally, chemical vapor deposition (CVD) is employed to fabricate structurally controlled materials (such as vertically aligned carbon nanotubes (VA-CNTs) and graphene) [36]. During the growth process, heteroatom-containing gases or carbon sources incorporating heteroatoms are introduced to achieve in situ doping [37]. Furthermore, hydrothermal/solvothermal methods enable simultaneous doping and reduction within sealed reactors. Mechanochemical approaches facilitate edge halogenation [38].

In this study, we proposed a universal synthesis strategy to obtain five-coordinated square pyramidal configurations through oxidative polymerization. Introducing strong electronegative heteroatoms (S and Cl) through axial coordination can alter the symmetry and modulate the electronic structure of conventional Fe–N_4_ configuration significantly. Due to the strong electronegativity of the introduced heteroatoms (Cl, S), the pronounced electron-withdrawing effect causes the axial electrons in the X–Fe (X = Cl, S) coordination bonds to become polarized toward the heteroatoms. This breaks the originally symmetric electronic structure of Fe, leading to increased activity in the trans-axial electrons [39, 40]. As shown in the table of contents (TOC) figure, chlorine with higher electronegativity provides Cl–Fe–N/C with a d-band center closer to the Fermi level and stronger coordination capability. Three SACs (Fe–N_4_, Cl–Fe–N_4_ and S–Fe–N_4_) were synthesized and their electrocatalytic performance toward ORR was systematically evaluated. Electrochemical measurements revealed that Cl–Fe–N_4_ outperformed S–Fe–N_4_ and other catalysts in catalytic activity and stability toward ORR under simulated seawater conditions. In alkaline seawater, Cl–Fe–N_4_ demonstrated excellent Cl^−^ selective repulsion properties and intrinsic activity. It achieved a remarkable limiting current density of 5.8 mA cm^−2^, significantly higher than S–Fe–N_4_ and commercial Pt/C, while maintaining superior half-wave and onset potentials. When applied as a cathode catalyst in SZABs, Cl–Fe–N_4_ demonstrated high power density and specific capacity, along with exceptional cycling stability over 200 h. Density functional theory (DFT) calculation results demonstrate that the introduction of Cl can effectively enhance the activity of reaction intermediates, increase the electron density of Fe active sites, and promote the overall ORR process. These findings not only deepen the understanding of heteroatom-doped SACs but also establish Cl–Fe–N_4_ as a superior candidate for high-performance SZABs, particularly highlighting the unique advantages of Cl doping in enhancing both activity and durability.

## Experimental Sections

### Chemicals and Reagents

1,5-diaminonaphthalene (97%, Aladdin), ethanol (99.7%, Shanghai Lingfeng), FeCl_3_ (99.9%, Macklin), NaClO (30 wt% available chlorine, Macklin), concentrated H_2_SO_4_ (95.0–98.0 wt%, Shanghai Lingfeng), (NH_4_)_2_S_2_O_8_ (98%, Macklin), H_2_O_2_ (30 wt%, Shanghai Lingfeng), isopropanol (99.9%, Shanghai Lingfeng), Nafion (5 wt%, Sigma-Aldrich), alumina powder (1, 0.3, and 0.03 μm, Shanghai Chuxi) and Pt/C catalyst (40 wt%, Hispec4000, Johnson Matthey) were used without further purification. Alkaline synthetic seawater was obtained by adding 1 M KOH to commercially simulated seawater (salinity 35%, Chuangfeng) until the pH value reached 13. All solutions were prepared using deionized water (18.2 MΩ cm, MilliQ).

### Synthesis of Materials

To prepare the catalyst, 1 g of 1,5-diaminonaphthalene was dissolved in 220 mL of ethanol. Subsequently, 24.3 mg of FeCl_3_ was dispersed in 20 mL of ethanol and added to the above solution, followed by stirring for 10 min. Next, 1 g of NaClO was dissolved in 10 mL of deionized water and introduced into the mixture. The resulting solution was continuously stirred at 25 °C for 22 h in a water bath. The solution was then transferred to an oven and heated at 80 °C for 4 h to facilitate oxidative polymerization, yielding the catalyst precursor powder. The precursor was then placed in an alumina combustion boat and subjected to pyrolysis in a tube furnace under an argon atmosphere. The temperature was increased at a rate of 10 °C min^−1^ to 950 °C and maintained for 2 h to obtain a high surface area catalyst. The pyrolyzed product was subsequently dispersed in 0.5 M H_2_SO_4_ and refluxed at 120 °C for 8 h with continuous stirring to remove excess metal. Finally, the product was filtered, thoroughly washed, and dried overnight at 60 °C to obtain the final catalyst. S–Fe–N_4_ and Fe–N_4_ were obtained using similar method by replacing NaClO with (NH_4_)_2_S_2_O_8_.

### Material Characterization

Da Vinci diffractometer (Bruker D8, Cu radiation) was used to obtain the X-ray diffraction (XRD) patterns. Thermo Scientific K-Alpha X-ray Photoelectron Spectrometer System (Al Kα, 1486.6 eV) was used to obtain the surface X-ray photoelectron spectra (XPS). An automatic fast surface and porosity analyzer (ASAP 2460, Micromeritics, America) was used to get the Brunauer − Emmett − Teller (BET) surface area. In addition, transmission electron microscopy (TEM, JEM-F200, JEOL, Japan) was used to observe the morphological characteristics of the samples. Furthermore, high-angle annular dark-field scanning transmission electron microscopy (HAADF-STEM, JEM-ARM 200F, JEOL) was employed to observe the dispersed single-atom imaging in the catalyst. The X-ray absorption spectra (XAS) including X-ray absorption near-edge structure (XANES) and extended X-ray absorption fine structure (EXAFS) of the samples (6900 to 7855 eV were collected at the Singapore Synchrotron Light Source (SSLS) center, where a pair of channel-cut Si (111) crystals was used in the monochromator). The storage ring was working at the energy of 700 MeV with an average electron current of below 200 mA. For reference, XAS spectra of iron(II) phthalocyanine (Fe^II^Pc), metallic iron foil, FeO, and Fe_2_O_3_ were also measured. The X-ray absorption near-edge structure and extended X-ray absorption fine structure spectra were processed using Athena software [41], including Fourier transform analysis within the k-range of 3–8 Å^−1^.

### Electrochemical Measurements

All electrochemical measurements were conducted using an electrochemical workstation (SP-300, Bio-Logic, France) unless otherwise stated, and carried out at 25 °C. Prior to use, all working electrodes were polished with 1, 0.3, and 0.03 μm alumina powder in sequence.

#### Rotating Disk Electrode Measurements

The linear sweep voltammetry (LSV) measurements and chronoamperometry were performed for different purposes using a three-electrode cell where a glassy carbon rotating disk electrode (RDE, 5 mm in diameter) was the working electrode, an Ag/AgCl electrode was the reference electrode, and a graphite rod electrode served the counter electrode. 7.36 mg of catalyst (Cl– Fe–N_4_, S–Fe–N_4_, Fe–N_4_) was dispersed in a mixed solution containing 240 μL isopropanol, 240 μL water, and 20 μL Nafion, and 0.735 mg of Pt/C catalyst was dispersed in the same mixed solution and sonicated for 30 min to obtain the catalyst ink. In addition, chronoamperometry (CA) was used to evaluate the resistance of the catalysts to Cl^−^ using the same settings as LSV but with a rotating speed of 1600 rpm and an applied constant overpotential of 0.57 V *vs.* RHE. High-purity oxygen was continuously introduced during testing to ensure a constant dissolved oxygen concentration in the electrolyte. A catalyst loading of 0.75 mg cm^−2^ was achieved by dropcasting 10 μL of ink onto the surface of the working electrode for both LSV and CA.

#### Seawater-based Zinc-air Battery Test

In the seawater-based zinc-air battery, polished zinc foil was used as the anode, and the cathode consists of nickel foam with catalyst-coated layers and gas diffusion layers on both sides. The catalyst layer is composed of catalyst, carbon black, and polytetrafluoroethylene (PTFE) in a mass ratio of 7:2:1, with a catalyst loading of 1 mg cm^−2^. The electrolyte was alkaline synthetic seawater (pH = 13) supplemented with 0.2 M Zn(CH_3_COO)_2_·2H_2_O. Power density curves were obtained using a electrochemical workstation (CHI 760E), while long-term galvanostatic charge/discharge cycling curves and specific capacity curves were acquired through a LAND battery testing system (CT2001A).

#### In situ Fourier Transform Infrared Spectroscopy

CA tests were conducted at applied potentials ranging from 0.1 to 0.9 V *vs.* RHE in alkaline synthetic seawater (pH = 13). The catalysts were dropcasted onto a Si crystal, with an Ag/AgCl electrode as the reference electrode and a platinum wire electrode as the counter electrode. A Fourier transform infrared spectrometer (Thermo Fisher Nicolet is50) equipped with an MCT detector was used for measurements at a resolution of 2 cm^−1^ and a wavelength range of 1000–2000 cm^−1^, with 32 repeated scans performed at each potential.

## Results and Discussion

### Material Characterization

**Fig. 1:**
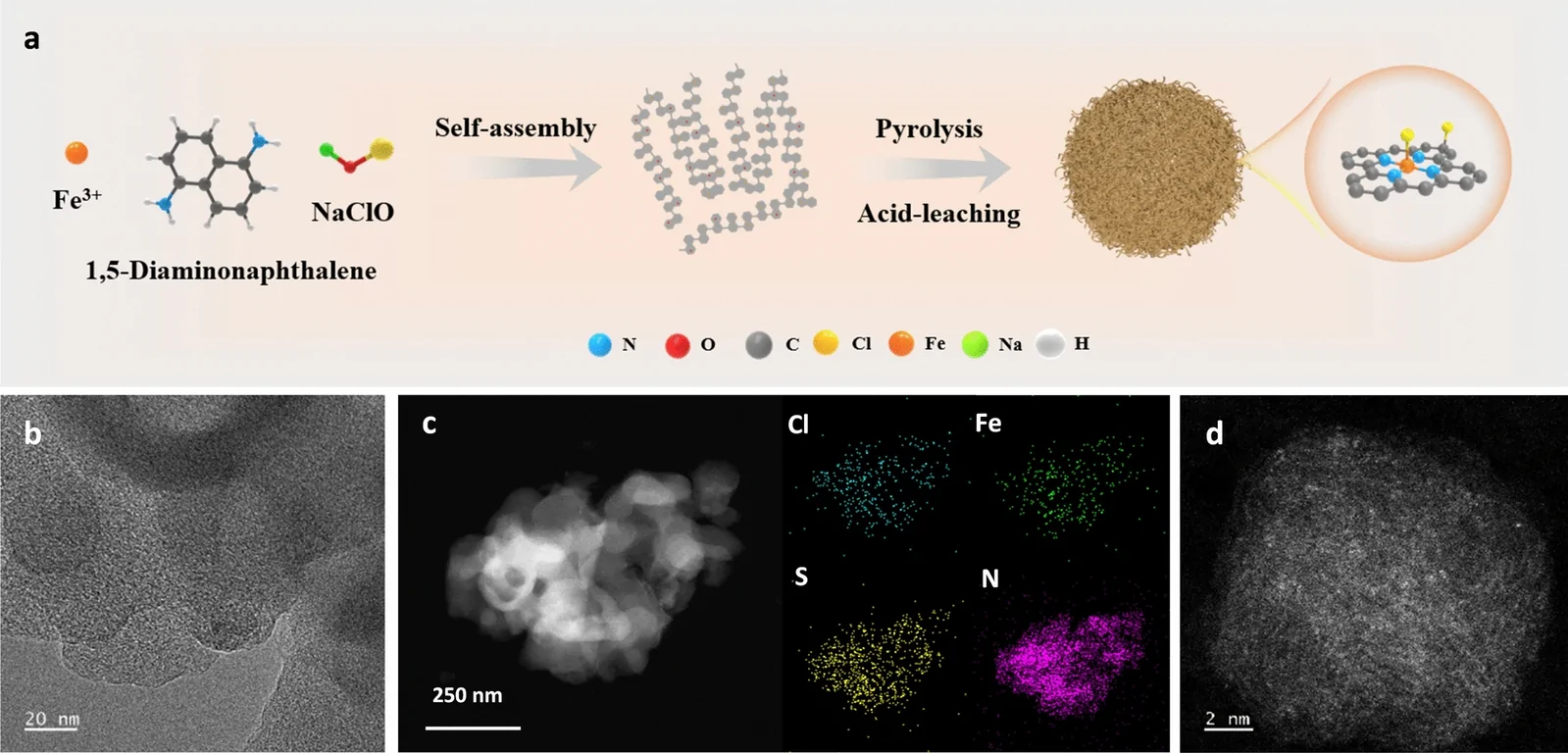
**a** Schematic diagram of synthesis process. **b** HRTEM pattern of Cl– Fe–N_4_. **c** STEM-EDS image of Cl– Fe–N_4_. **d** HAADF-STEM mapping of Cl– Fe–N_4_

The catalyst precursor was obtained through a one-pot synthesis method, and then, the catalyst products (Cl– Fe–N_4_, S–Fe–N_4_, and Fe–N_4_) with high specific surface area were obtained through pyrolysis and acid leaching. The detailed synthesis procedure is illustrated in Fig. 1a.

**Fig. 2:**
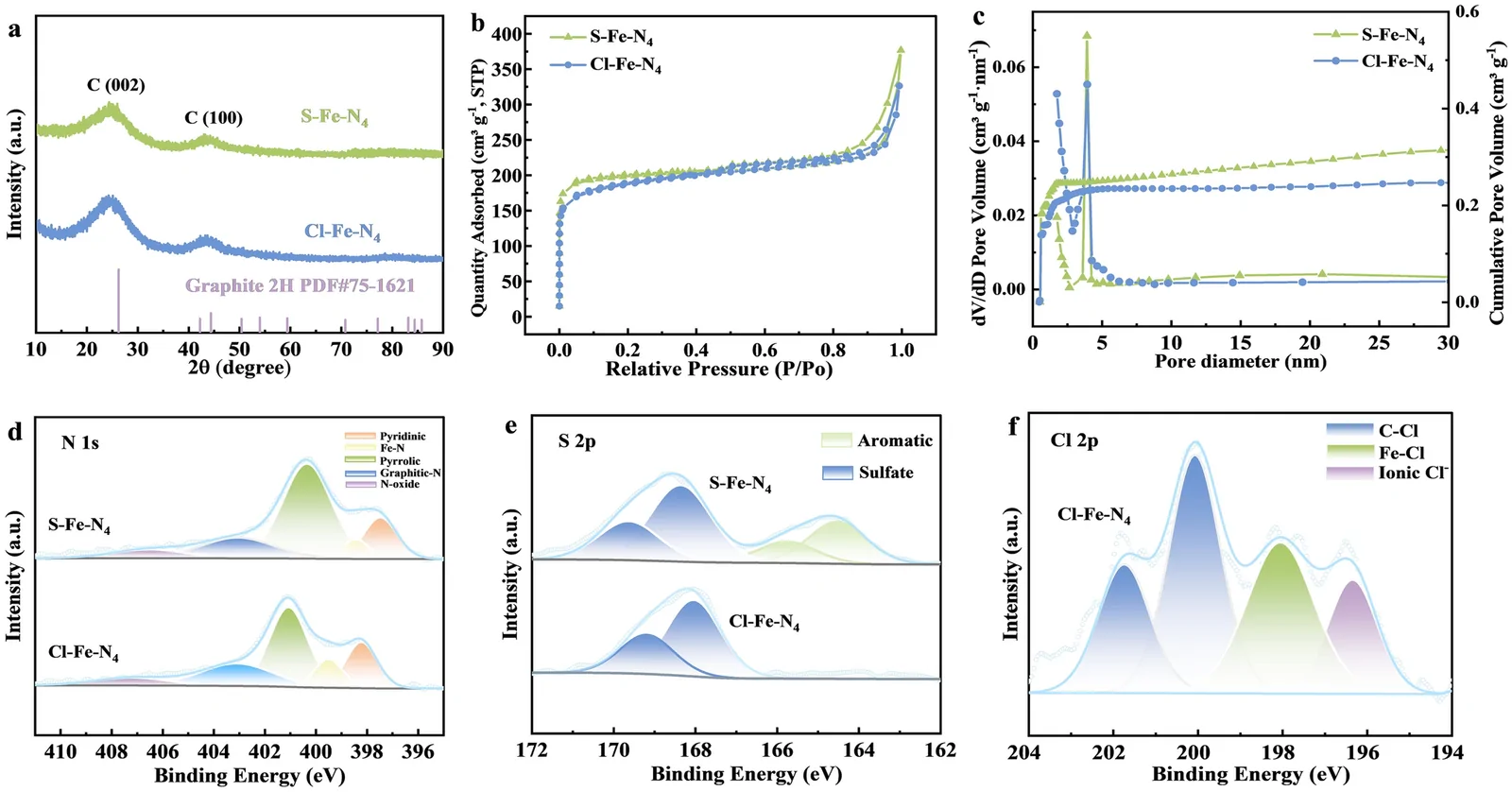
**a** XRD patterns of Cl–Fe–N_4_ and S–Fe–N_4_. **b** N_2_ adsorption–desorption isotherms of Cl–Fe–N_4_ and S–Fe–N_4_ at 77 K. **c** Pore size distribution curves and cumulative pore volume curves of Cl–Fe–N_4_ and S–Fe–N_4_. Deconvoluted high-resolution XPS spectra of **d** N 1*s* and **e** S 2*p* for Cl–Fe–N_4_ and S–Fe–N_4_, and **f** Cl 2*p* for Cl– Fe–N_4_

**Fig. 3:**
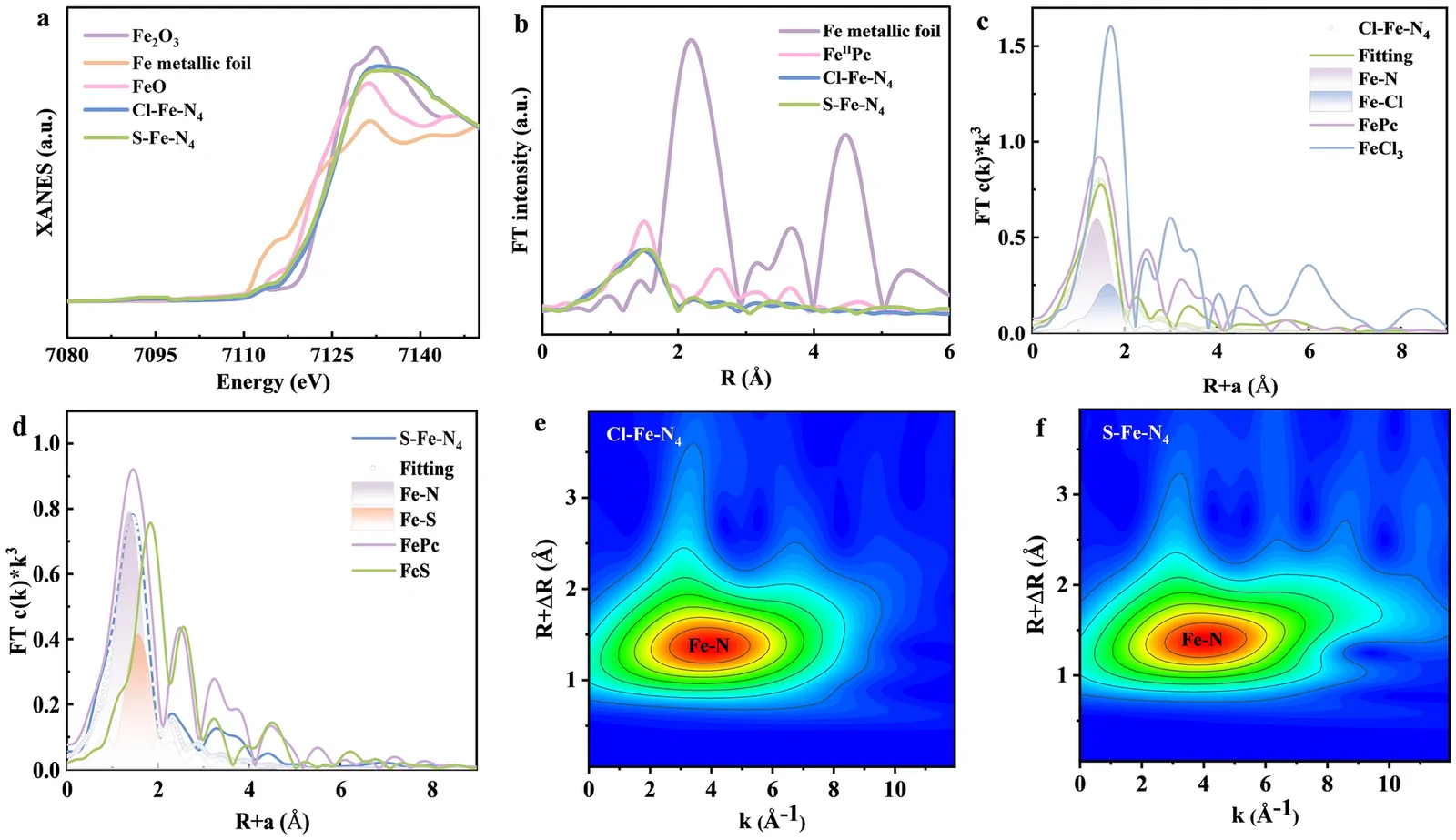
**a** X-ray absorption near-edge structure and **b** Fourier-transformed extended X-ray absorption fine structure curves of Cl– Fe–N_4_, S–Fe–N_4_ and reference samples at Fe k-edge. k3-weighted Fe k-edge EXAFS fitting curves of **c** Cl–Fe–N_4_, **d** S–Fe–N_4_ and reference samples in k space. Wavelet transform EXAFS analysis of the Fe k-edge for **e** Cl–Fe–N_4_ and **f** S–Fe–N_4_

The morphology of all synthesized materials was characterized using TEM. Amorphous carbon structures were observed for Cl–Fe–N_4_ (Fig. 1b) and S–Fe–N_4_ (Fig. S1), without nanoparticles or clusters, and scanning transmission electron microscopy − energy-dispersive X-ray spectroscopy (STEM-EDS) mappings (Fig. 1c) revealed uniformly dispersed Fe and Cl elements. In contrast, Fe_2_O_3_ nanoparticles were identified in Fe–N_4_ (Fig. S1), where the observed lattice spacing of 0.22 nm corresponds to the (311) crystal plane of Fe_2_O_3_. Additionally, graphitized carbon structures were present in Fe–N_4_, with a lattice spacing of 0.34 nm corresponding to the (002) plane of graphitized carbon, and aggregated Fe elements were also observed in its STEM-EDS mapping. Figures 1d and S1 display high-resolution HAADF-STEM images of Cl–Fe–N_4_ and S–Fe–N_4_, demonstrating the presence of atomically dispersed Fe atoms. Furthermore, the crystal structures of the catalysts were determined using XRD. Figure 2a shows that Cl–Fe–N_4_ and S–Fe–N_4_ exhibit two broad peaks at 25° and 44°, corresponding to the amorphous carbon structure observed in the TEM images, while Fe–N_4_ displays a highly crystalline graphitic C (002) Bragg reflection at 25° (Fig. S2), indicating the existence of graphitized carbon, consistent with TEM observations. Subsequently, the porosity and specific surface area of catalysts were studied through N_2_ adsorption–desorption measurements. The N_2_ adsorption–desorption curves (Fig. 2b) indicated that Cl–Fe–N_4_ and S–Fe–N_4_ demonstrated a typical Type IV isotherm with H4 hysteresis loop and the former has a high specific surface area of 708.0 m^2^ g^−1^, while S–Fe–N_4_ shows a slightly higher surface area of 753.7 m^2^ g^−1^. Based on the DFT model-derived pore volume increment curve (Fig. S3), cumulative pore volume curve and pore size distribution curve (Fig. 3c), the analysis reveals that the pore volume increase in both Cl–Fe–N_4_ and S–Fe–N_4_ is primarily concentrated within the 0–1 nm range. Specifically, Cl–Fe–N_4_ exhibits a micropore proportion of 63.0%, mesopores of 13.1%, and macropores of 23.9%. In contrast, S–Fe–N_4_ demonstrates a micropore proportion of 47.9%, mesopores of 18.2%, and macropores of 33.8%. Among them, the micropores are likely attributed to the formation of gases from Cl- and S-containing functional groups in the material during the pyrolysis process, while the mesopores and macropores may originate from the removal of surface nanoparticles in the acid leaching process. The higher proportion of micropores in Cl–Fe–N_4_ facilitates the exposure of more active sites and promotes the uniform dispersion of Fe atoms, thereby increasing the number of active sites [42]. The confinement effect of micropores enhances the adsorption strength of O_2_ molecules at active sites during the ORR, while stabilizing key intermediates in the reaction process, ultimately reducing the reaction energy barrier [43, 44].

The surface chemistry and bonding configuration of the catalysts were investigated using XPS. In the XPS survey spectrum, distinct C 1*s*, N 1*s*, and O 1*s* peaks can be observed at around 285, 401, and 532 eV, respectively, indicating their relatively high content in the three catalysts. In the high-resolution N 1*s* spectra (Fig. 2d), peaks located at 398.3, 399.7, 401.1, 402.9, and 406.8 eV can be assigned to pyridinic-N, Fe–N coordination, pyrrolic-N, graphitic-N, and N-oxide species, respectively [45, 46]. It can be observed that the binding energies of some N species in Cl–Fe–N_4_ are slightly higher than those in S–Fe–N_4_, which may be due to the electronegativity difference. As Cl is more electronegative than S, when coordinated axially to Fe, it withdraws electron density from the Fe center. This electron withdrawal is then propagated through the Fe–N bonds to the surrounding N atoms, resulting in a slight decrease in electron density on the N atoms. Consequently, this leads to a higher binding energy for the N species in Cl–Fe–N_4_ compared to S–Fe–N_4_. For the S 2*p* spectra (Fig. 2e), Cl–Fe–N_4_ exhibits only one dominant peak at around 168 eV, attributed to sulfate species introduced during sulfuric acid leaching treatment [45, 46]. In contrast, S–Fe–N_4_ shows an additional peak at ~ 164 eV, corresponding to aromatic sulfur/sulfones and thiophene structures formed through ammonium persulfate-promoted oxidative polymerization of diaminonaphthalene monomers during the hydrothermal reaction [45]. Previous reports have shown that such heteroatoms introduction onto carbon-based support materials helps to precisely regulate electronic states and local charge densities, breaking the Fe symmetric electronic structure to fine-tune the adsorption and reactivity of microporous polymers, enhancing charge transfer between catalysts and O*, and adsorption of electrophilic oxygen intermediates [47–49]. In the Cl 2*p* deconvoluted spectrum of Cl–Fe–N_4_ (Fig. 2f), the peak at 198 eV is identified as Fe–Cl species [50] while another peak at 200 eV corresponds to organic C–Cl bonds [50]. This dual observation confirms that Cl atoms not only bond to carbon sites but also participate in Fe coordination, indicating the formation of Cl–Fe–N_4_ coordination structure. Furthermore, comparative analysis of high-resolution Fe 2*p* spectra (Fig. S4) shows that the position of Fe^2+^ species in Cl–Fe–N_4_ is significantly negatively shifted, while the binding energy of Fe^3+^ species is positively shifted, indicating that Cl doping effectively changes the electronic structure of Fe. The axial coordination of Cl injects electrons into the 3*d* orbitals of Fe^2^⁺, increasing its electron density (as confirmed by Bader charge analysis in Fig. 5b in the theoretical calculation sections, showing electron transfer from Cl to Fe). This elevated electron density weakens the binding energy of the Fe 2*p* orbitals, resulting in a shift of the Fe^2^⁺ binding energy toward lower energy (negative shift) [39]. This process stabilizes the low oxidation state of Fe^2^⁺ and optimizes its adsorption capacity for oxygen-containing intermediates, consistent with DFT calculations (Fig. 5c). In contrast, Fe^3^⁺ inherently possesses a high oxidation state. The strong electron-withdrawing effect of Cl further depletes its electrons, reducing the electron density of Fe^3^⁺. This decreased electron density strengthens the binding energy of the Fe 2*p* orbitals, manifesting as a shift toward higher energy (positive shift) [39, 40]. Such modulation facilitates electron acceptance by Fe^3^⁺, promoting the reduction of *O_2_ to *OOH during the ORR process. Additionally, the atomic ratios of different elements in the samples were quantified based on XPS data (Table S1), and the relative contents of nitrogen species were systematically analyzed (Table S2). Among them, the nitrogen content in Cl–Fe–N_4_ (~ 4.73%) is relatively higher compared to that in S–Fe–N_4_ (~ 4.21%) and Fe–N_4_ (~ 3.55%), which is beneficial for anchoring more Fe atoms and forming additional active sites.

To further investigate the local coordination environment and valence state of Fe in Cl–Fe–N_4_ and S–Fe–N_4_, XAS was performed, yielding their XANES and EXAFS spectra. The XANES spectra of Cl–Fe–N_4_ and S–Fe–N_4_ (Fig. 3a) show that the Fe k-edge absorption energies lie between FeO and Fe_2_O_3_, indicating an intermediate valence state between + 2 and + 3. Notably, Cl–Fe–N_4_ exhibits a slightly higher valence state than S–Fe–N_4_, possibly due to the shortening of the Fe–N bond length from 2.02 Å in S–Fe–N_4_ to 1.91 Å after Cl modification [51]. The high electronegativity of Cl may induce D-orbital contraction in Fe, enhancing the covalency of the Fe–N bonds, thereby requiring a higher oxidation state of Fe to balance the charge. The EXAFS spectra of Fe foil (Fig. 3b) display a dominant Fe–Fe scattering peak at 2.2 Å. In contrast, neither Cl–Fe–N_4_ nor S–Fe–N_4_ shows significant signals at this position, but instead exhibits a prominent Fe–N scattering peak at 1.45 Å, consistent with the main peak position of iron(II) phthalocyanine (FeᴵᴵPc). This further confirms the atomic dispersion of Fe as Fe–N single-atom sites in the samples. EXAFS fitting results reveal that Cl–Fe–N_4_ has an Fe–Cl bond length of 2.17 Å, matching the bond lengths in FeCl_4_^−^ tetrahedra (~ 2.17–2.19 Å). This is characteristic of monodentate Cl ligands, with Cl axially adsorbed on Fe–N_4_ to form a five-coordinated square pyramidal structure (Figs. 3c and S5, Table S3). For S–Fe–N_4_, the Fe–S bond length is 2.67 Å, larger than typical Fe–S coordination bonds (2.2 – 2.5 Å), suggesting weaker coordination or steric hindrance when S acts as an axial ligand. This configuration also adopts a five-coordinated square pyramidal structure (Figs. 3d and S5, Table S3). Wavelet transform (WT) EXAFS analysis of the Fe k-edge (Figs. 3e, f and S5) further confirms the absence of Fe–Fe metallic bonding in Cl–Fe–N_4_ and S–Fe–N_4_. Instead, distinct Fe–N coordination signals at ~ 1.4 Å are observed, consistent with the WT patterns of FeᴵᴵPc.

### Evaluation of Electrocatalytic Performance toward ORR

**Fig. 4:**
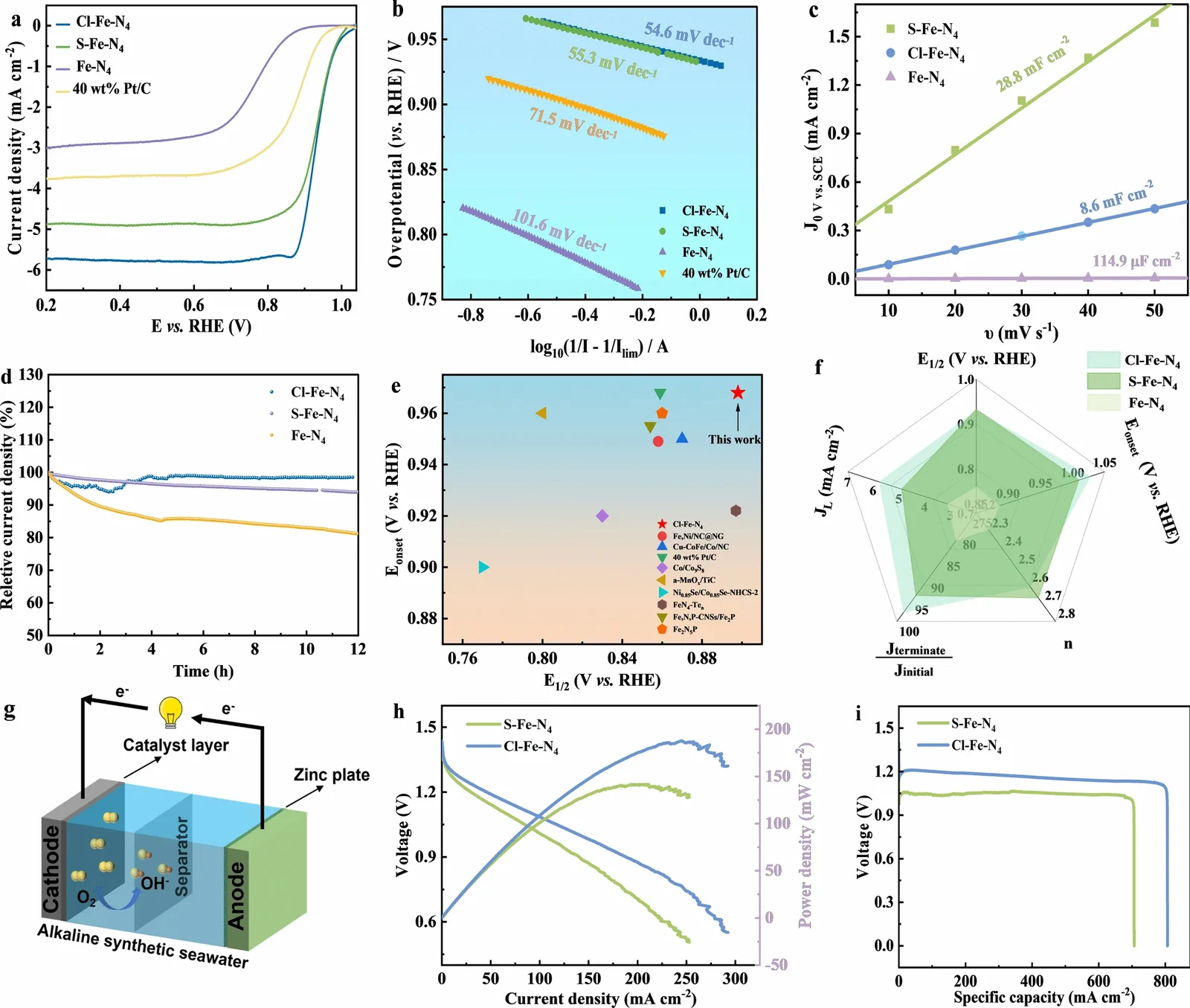
**a** LSV polarization curves and** b** the corresponding mass transport-corrected Tafel plots of different catalysts using RDE at 1600 rpm in alkaline synthetic seawater (pH = 13) with a scan rate of 10 mV s^−1^. Catalyst loading: 0.75 mg cm^−2^, and 40 wt% Pt/C: 0.075 mg cm^−2^. **c** Normalized C_dl_ of different catalysts in alkaline synthetic seawater. **d** Chronoamperometry curves of different catalysts using RDE at 0.57 V *vs.* RHE in alkaline synthetic seawater at 1600 rpm. **e** Comparison of ORR performance between catalysts in this work and previously reported catalysts with respect to E_1/2_ and E_onset_. **f** Comprehensive performance comparison of catalysts in this work in alkaline synthetic seawater. **g** Schematic diagram of seawater-based zinc-air battery. **h** Polarization and power density curves and **i** specific capacity of Cl–Fe–N_4_ and S–Fe–N_4_ at 10 mA cm.^−2^

The ORR performance of the catalysts was evaluated via LSV using a RDE at 1600 rpm in O_2_-saturated 0.1 M KOH at a scan rate of 10 mV s^−1^ to compare the electrocatalytic activity of these catalysts. As shown in Fig. S6, Cl–Fe–N_4_ demonstrated superior catalytic activity with an onset potential (E_onset_) of 0.968 V *vs.* RHE at a current density of 0.1 mA cm^−2^, an E_1/2_ of 0.898 V, and a limiting current density (J_L_) of 5.61 mA cm^−2^ among all synthesized catalysts. It is worth noting that the performance of Cl–Fe–N_4_ is better than commercial 40 wt% Pt/C (E_onset_ = 0.968 V, E_1/2_ = 0.859 V, J_L_ = 5.06 mA cm^−2^). Additionally, based on comparisons of onset potential and half-wave potential, Cl–Fe–N_4_ demonstrates superior ORR catalytic activity in 0.1 M KOH compared to most reported ORR catalysts to date (Fig. 4e) [52–59]. This demonstrates that the adsorption of Cl at the axial position in the traditional Fe–N_4_ structure forms a Cl–Fe–N_4_ configuration, which effectively optimizes the electronic structure of Fe, facilitates the adsorption of O_2_ molecules, and significantly enhances the ORR activity.

To assess practical applicability in Cl^−^-rich environments, LSV tests were performed in 1) 0.1 M KOH with 0.5 M KCl and 2) alkaline synthetic seawater (pH = 13), respectively. In 0.1 M KOH with 0.5 M KCl (Fig. S6), Cl–Fe–N_4_ maintained a high E_onset_ of 1.000 V and E_1/2_ of 0.950 V, with a minor decrease in J_L_ to 4.75 mA cm^−2^, surpassing S–Fe–N_4_ (E_1/2_ = 0.927 V), Fe–N_4_ (E_1/2_ = 0.711 V), and Pt/C (E_1/2_ = 0.878 V). Furthermore, in synthetic alkaline seawater, the E_1/2_ of Cl–Fe–N_4_ and S–Fe–N_4_ reached 0.931 V and 0.933 V, respectively, both significantly higher than those of undoped Fe–N_4_ (E_1/2_ = 0.758 V) and Pt/C (E_1/2_ = 0.876 V) (Fig. 4a). This demonstrates that heteroatom doping significantly enhances the ORR catalytic performance while exhibiting higher resistance to Cl^−^ poisoning. This is attributed to the Cl- and S- coordination structure inducing a highly negatively charged Fe active center, which enables highly selective repulsion toward Cl^−^ in seawater, thereby preventing the poisoning of active sites. Analysis of the ORR electron transfer number (n) using the Koutecký–Levich (K-L) equation revealed that Cl–Fe–N_4_ exhibits average n of 4.06 and 4.02 at varying potentials in 0.1 M KOH and 0.1 M KOH with 0.5 M KCl, respectively (Fig. S7), suggesting that the vast majority of its active sites maintain a highly efficient four-electron pathway even under high Cl^−^ concentrations. In contrast, in high Cl^−^ electrolytes, the n of S–Fe–N_4_ and Fe–N_4_ catalysts decreased significantly to 2.97 and 2.24, respectively (Fig. S7). This demonstrates that the Cl–Fe–N_4_ coordination structure effectively enables active sites to resist Cl^−^ poisoning. Mass transport-corrected Tafel analysis [60] was further conducted to get kinetics information where in alkaline synthetic seawater, as shown in Fig. 4b, Cl–Fe–N_4_ and S–Fe–N_4_ exhibited relatively small Tafel slopes of 54.6 and 55.3 mV dec^−1^, respectively, while Pt/C and Fe–N_4_ showed significantly larger Tafel slopes of 71.5 and 101.6 mV dec^−1^, respectively. The smallest Tafel slope of Cl–Fe–N_4_ catalyst further illustrated the excellent electrocatalytic activity toward ORR. In addition, the double-layer capacitance (C_dl_) was investigated to evaluate the electrochemical active surface area (ECSA) of the samples. CV measurements at different scan rates were performed in the non-Faradaic region of the ORR in alkaline synthetic seawater (Fig. S8) to determine the C_dl_ values where S–Fe–N_4_ and Cl–Fe–N_4_ exhibited the C_dl_ of 28.8 and 8.6 mF cm^−2^, respectively, higher than Fe–N_4_ (Fig. 4c). The relatively high value of C_dl_ in S–Fe–N_4_ and Cl–Fe–N_4_ ensured a faster charge transfer which therefore enhanced the electrocatalytic performance toward ORR.

Stability is also a crucial indicator for a seawater catalyst. CA tests (Fig. 4d) were conducted in alkaline seawater at an overpotential of 0.57 V *vs.* RHE using a RDE modified with Fe–N_4_, S–Fe–N_4_, and Cl– Fe–N_4_. Both Cl–Fe–N_4_ and S–Fe–N_4_ demonstrated excellent stability after a 12-h CA test, retaining 96.7% and 94.0% of their initial current density (Fig. 4d), respectively. The E₁/_2_ of these samples decreased by only 7 and 8 mV after testing (Fig. S9). In contrast, the undoped Fe–N_4_ exhibited poor stability in synthetic seawater, within its final current density dropping to 81.3% of the initial value and a significant E₁/_2_ loss of 43.5 mV (Fig. S9). These results further confirm the outstanding resistance of heteroatom-doped catalysts to seawater-induced poisoning of active sites.

We assembled alkaline synthetic seawater-based Zn-air batteries using Cl–Fe–N_4_ and S–Fe–N_4_ with excellent ORR performance as the catalyst coatings for the air cathode (Fig. 4g). The results showed that under identical discharge current densities, the Cl–Fe–N_4_-based SZAB exhibited higher discharge voltage than the S–Fe–N_4_-based counterpart, along with superior discharge power density which achieved 187.7 mW cm^−2^ at a high current density of 245.1 mA cm^−2^, outperforming the S–Fe–N_4_-based SZAB (141.6 mW cm^−2^ at 201.7 mA cm^−2^) (Fig. 4h). At 10 mA cm^−2^, the Cl– Fe–N_4_-based SZAB demonstrated a specific discharge capacity of 806.5 mAh g^−1^, compared to 706.7 mAh g^−1^ for the S–Fe–N_4_-based SZAB (Fig. 4i). As shown in Fig. S10, galvanostatic charge–discharge cycling tests at 10 mA cm^−2^ further revealed that Cl–Fe–N_4_ maintained a more stable performance than S–Fe–N_4_ in alkaline seawater environments. These findings validate the exceptional ORR catalytic performance of both Cl–Fe–N_4_ and S–Fe–N_4_ in SZABs, highlighting their potential as cathode catalyst coatings.

To investigate the reasons for the degradation of catalyst performance in zinc-air batteries, Cl–Fe–N_4_ was characterized after battery tests. No significant morphological changes were observed in the SEM images before and after the test (Fig. S11), indicating no structural collapse of the catalyst. EDS mapping also showed no aggregation of Fe elements. Subsequent XPS analysis revealed that the peak positions of Fe 2*p*, Cl 2*p*, and N 1*s* remained basically unchanged (Fig. S12). However, the O 1*s* peak exhibited a slight positive shift, and the atomic ratio of oxygen increased from 19.46% to 28.45% (Table S4). This suggests that electrochemical oxidation may have occurred on the surface during the ORR process, covering active sites, or that strongly adsorbed ORR intermediates remained trapped at active sites, hindering further reactions, which casued the performance degradation.

### Mechanism Study via In situ FTIR and DFT Calculations

**Fig. 5:**
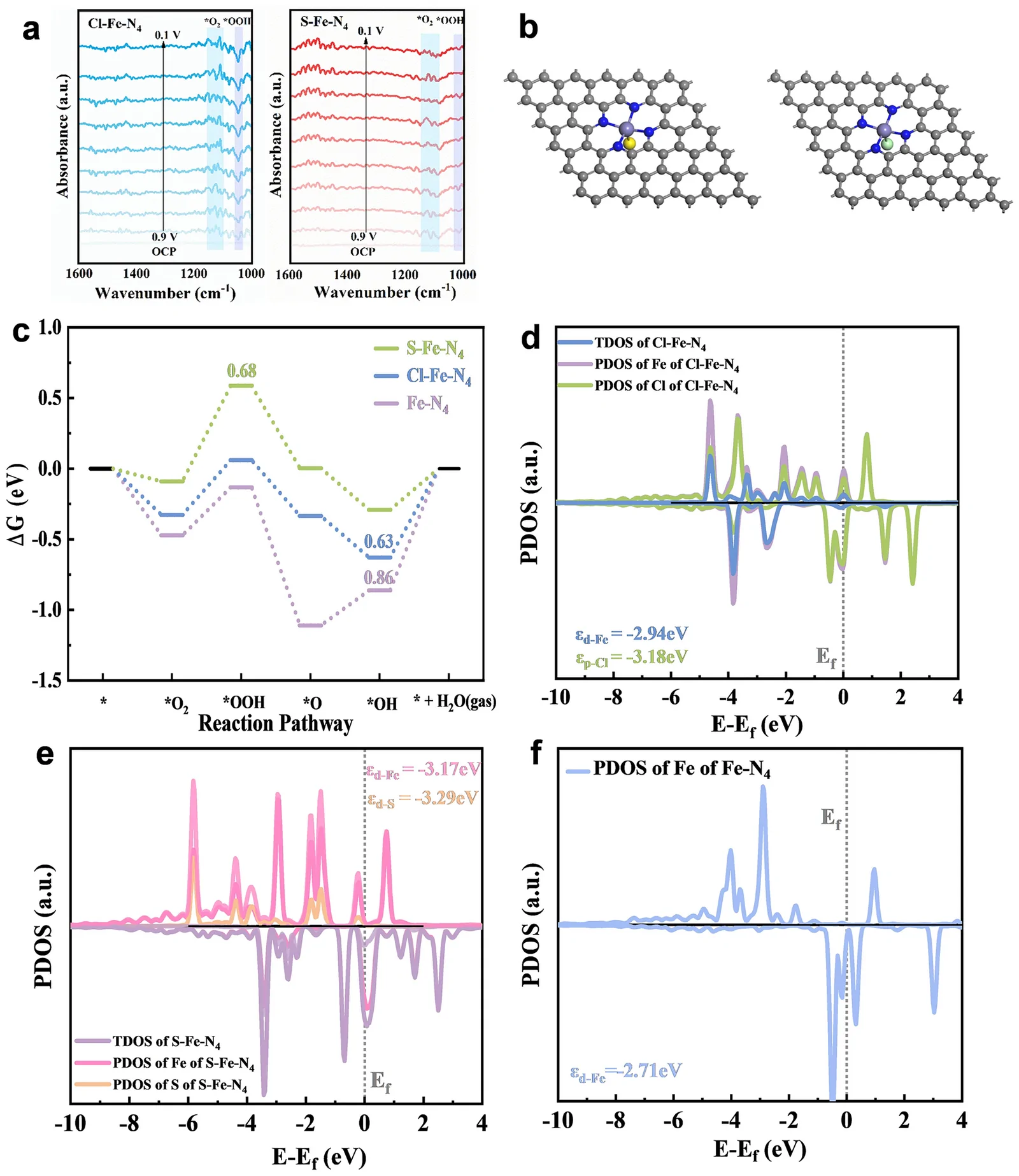
**a** In situ Fourier transform infrared spectroscopy of Cl–Fe–N_4_ and S–Fe–N_4_ for ORR. **b** Bader charge of Fe–N_4_, Cl–Fe–N_4_ and S–Fe–N_4_. Purple ball: Fe; green ball: Cl; yellow ball: S; gray ball: C; blue ball: N; red ball: O; white ball: H. **c** ORR reaction pathway on Fe–N_4_, S–Fe–N_4_ and Cl– Fe–N_4_. The projected density of states (PDOS) of **d** Fe–N_4_, **e** S–Fe–N_4,_ and **f** Cl–Fe–N_4_

To investigate the intermediate formation of catalysts during the ORR process, in situ Fourier transform infrared spectroscopy (FTIR) characterization of Cl–Fe–N_4_ and S–Fe–N_4_ was conducted in alkaline synthetic seawater under applied potentials ranging from 0.1 to 0.9 V vs. RHE. The results are presented in Fig. 5a. Notably, Cl–Fe–N_4_ exhibited distinct peaks at approximately 1100 cm^−1^ (assigned to *O_2_ formation) and 1046 cm^−1^ (assigned to *OOH consumption) at a low overpotential of 0.9 V. In contrast, S–Fe–N_4_ only showed a pronounced *O_2_ consumption peak near 1100 cm^−1^, with no detectable *OOH-related signals. This observation suggests that the subsequent reaction steps on S–Fe–N_4_ are hindered, leading to low pathway activity and causing the proton-coupled electron transfer (PCET) step to become the rate-determining step (RDS), which is consistent with DFT theoretical calculations. Conversely, Cl–Fe–N_4_ demonstrates a complete and efficient ORR pathway in alkaline seawater.

In order to further investigate the underlying mechanism of ORR reaction enhancement after axial coordination of heteroatoms on Fe–N_4_, DFT calculations were performed. Charge distribution of the as-prepared catalysts was first investigated and is presented in Figs. 5b and S16. Electrons are found to transfer from axially coordinated Cl and S to the Fe SA, increasing the electron density around Fe, which facilitates the ORR process. Therefore, by introducing axial S and Cl coordination, the electronic center of Fe SA is adjusted, making the reaction intermediates more active. Additionally, the electron density at the Fe SA sites is increased, enabling easier reduction in adsorbed intermediates, ultimately enhancing overall ORR activity. The mechanism of ORR was investigated for Fe–N_4_, S–Fe–N_4_, and Cl– Fe–N_4_, as shown in Fig. 5c. It was found that for Fe SA, the RDS in the ORR process is the formation of *H_2_O from *OH, with a free energy change (ΔG) of 0.86 eV, which limits its ORR activity. By introducing Cl and S atoms to form S–Fe SA and Cl–Fe SA, it was observed that the d-band center of single-atom Fe shifted from − 2.71 eV in Fe–N_4_ to − 2.94 and − 3.17 eV in the Cl–Fe SA and S-Fe SA, respectively, as shown in Fig. 5d-f. This shift reduced the adsorption energy of reaction intermediates, making the ORR process more favorable. Moreover, in Cl–Fe SA, the energy consumption for the step of *OH forming *H_2_O decreased to 0.63 eV, significantly enhancing its ORR activity. For S–Fe SA, the rate-determining step switched to the formation of *OOH from *O_2_, with energy consumption reduced to 0.68 eV, similarly improving ORR activity. Additionally, the adsorption configuration diagram in Fig. S17 revealed that the Cl–Fe–N/C structure maintains a relatively weak adsorption energy toward Cl^−^ ions at -0.28 eV, which was lower than its adsorption energy for O_2_. Consequently, the formed structure preferred to adsorb oxygen to facilitate the ORR process rather than re-adsorb Cl^−^ ions, demonstrating enhanced stability against chloride poisoning.

## Conclusions

In summary, this study successfully proposed an effective synthetic strategy for constructing Fe–N_4_ single-atom seawater catalysts with heteroatom axial coordination, and the XAS characterization results confirmed the five-coordinated square pyramidal structure. The electrochemical measurement showed that Cl–Fe–N_4_ exhibited the highest current density of 5.8 mA cm^−2^ in alkaline seawater compared to S–Fe–N_4_ (2.9 mA cm^−2^) and commercial Pt/C catalysts (3.0 mA cm^−2^), indicating that axial coordination of Cl atom enhanced the intrinsic activity of the material. Furthermore, Cl–Fe–N_4_ also maintained performance stability for 12 h in a three-electrode cell and the SZAB composed of Cl–Fe–N_4_ exhibited excellent peak power density (187.7 mW cm^−2^) at a high current density (245.1 mA cm^−2^) with a cycling stability for up to 200 h, demonstrating its ability to effectively repel Cl^−^ selectively in seawater and to reduce the performance degradation caused by catalyst structure degradation. DFT calculation results demonstrate that the introduction of Cl atoms can effectively increase the electron density of Fe SA active sites and promote the reduction in intermediates. These findings highlight the critical role of heteroatom coordination in marine catalyst design strategies, contributing to the advancement of efficient and durable catalysts for sustainable energy technologies.

## Supplementary Information

Below is the link to the electronic supplementary material.

Supplementary file1 (DOCX 3823 KB)
