# Supplementary material for: Heteroatom-Coordinated Fe–N4 Catalysts for Enhanced Oxygen Reduction in Alkaline Seawater Zinc-Air Batteries
Source: Nanomicro Lett. 2026 Jan 3;18:96. doi: 10.1007/s40820-025-01943-6 (PMC12759028; doi:10.1007/s40820-025-01943-6)
Supplement: Supplementary file 1 — Supplementary file1 (DOCX 3823 KB) [file 40820_2025_1943_MOESM1_ESM.docx]

Supporting Information for

**Heteroatom-Coordinated Fe-N₄ Catalysts for Enhanced Oxygen Reduction in Alkaline Seawater Zinc-Air Batteries**

Wenhan Fang^3,4^, Kailong Xu^1,2^, Xinlei Wang^1,2^, Yuanhang Zhu^5^, Xiuting Li^5^, Hui Liu^1,2^, Danlei Li^3, 4*^, Jun Wu^1, 2*^

^1^Department of School of Metallurgy and Environment, Central South University, Changsha 410083, P. R. China

^2^State Key Laboratory of Advanced Metallurgy for Non-ferrous Metals, Changsha 410083, P. R. China

^3^Department of Chemistry and Materials Science, School of Science, Xi’an Jiaotong-Liverpool University, Suzhou 215123, P. R. China

^4^Department of Chemistry, University of Liverpool L69 7ZD, the United Kingdom

^5^Institute for Advanced Study, Shenzhen University, Shenzhen 518060, P. R. China

*Corresponding authors. E-mail: [danlei.li@xjtlu.edu.cn](mailto:danlei.li@xjtlu.edu.cn) (Danlei Li); [wujun@csu.edu.cn](mailto:wujun@csu.edu.cn) (Jun Wu)

**Supplementary Figures**


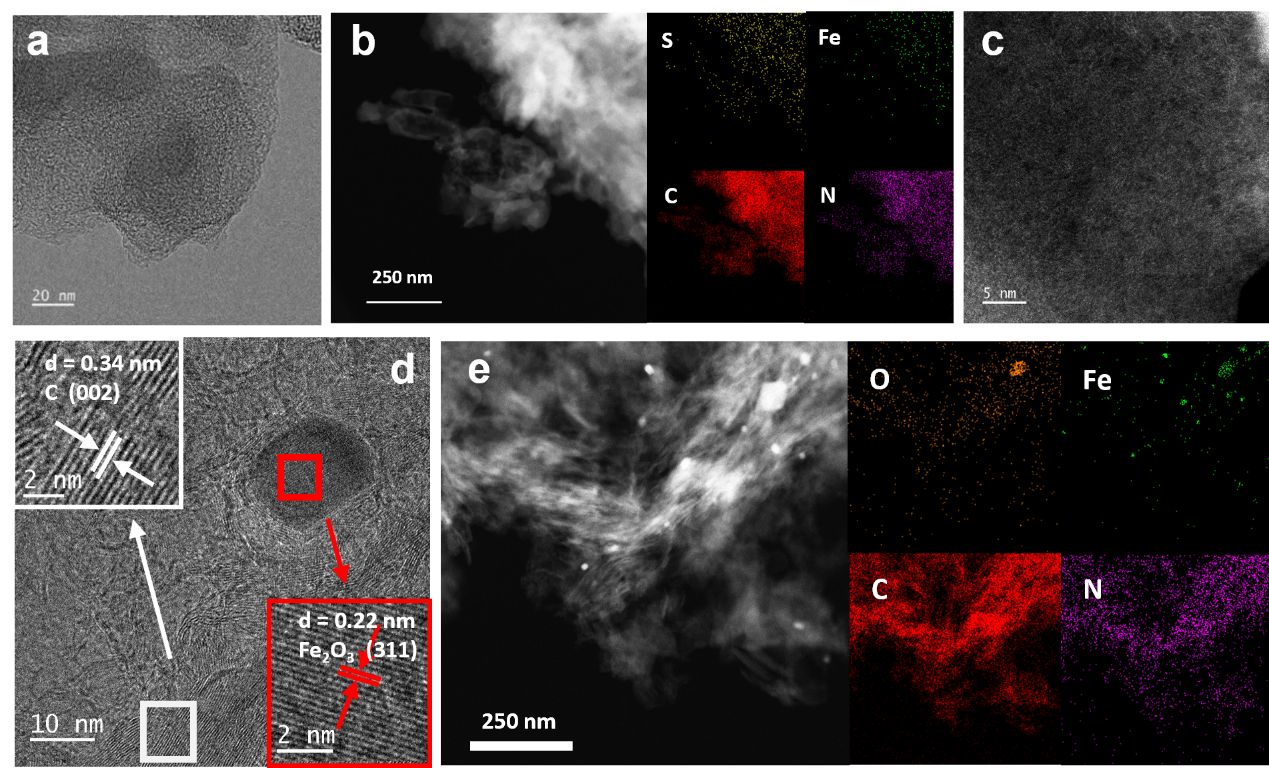


**Fig. S1** HRTEM pattern of **a**) S-Fe-N_4_, **d**) Fe-N_4_. STEM-EDS mapping of **b**) S-Fe-N_4_, **e**) Fe-N_4_. **c**) HAADF-STEM image of S-Fe-N_4_


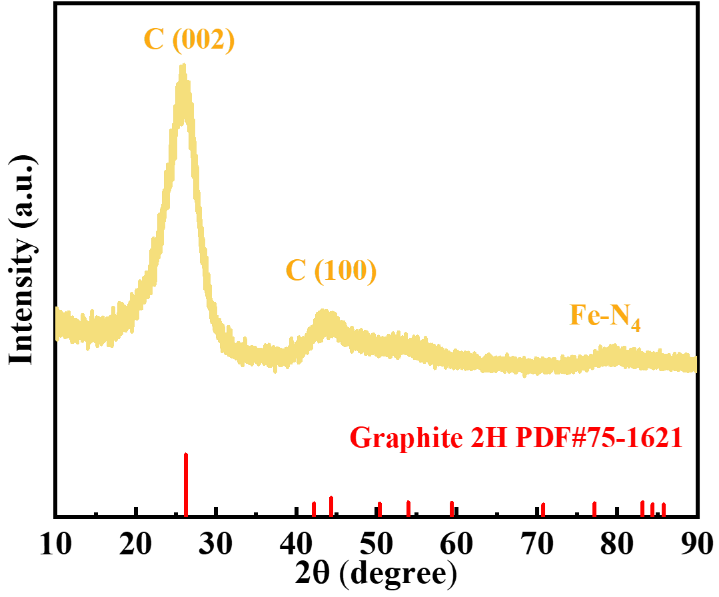


**Fig.** **S2** XRD pattern of Fe-N_4_

**Fig.** **S3** Pore volume increment curves of Cl-Fe-N_4_ and S-Fe-N_4_


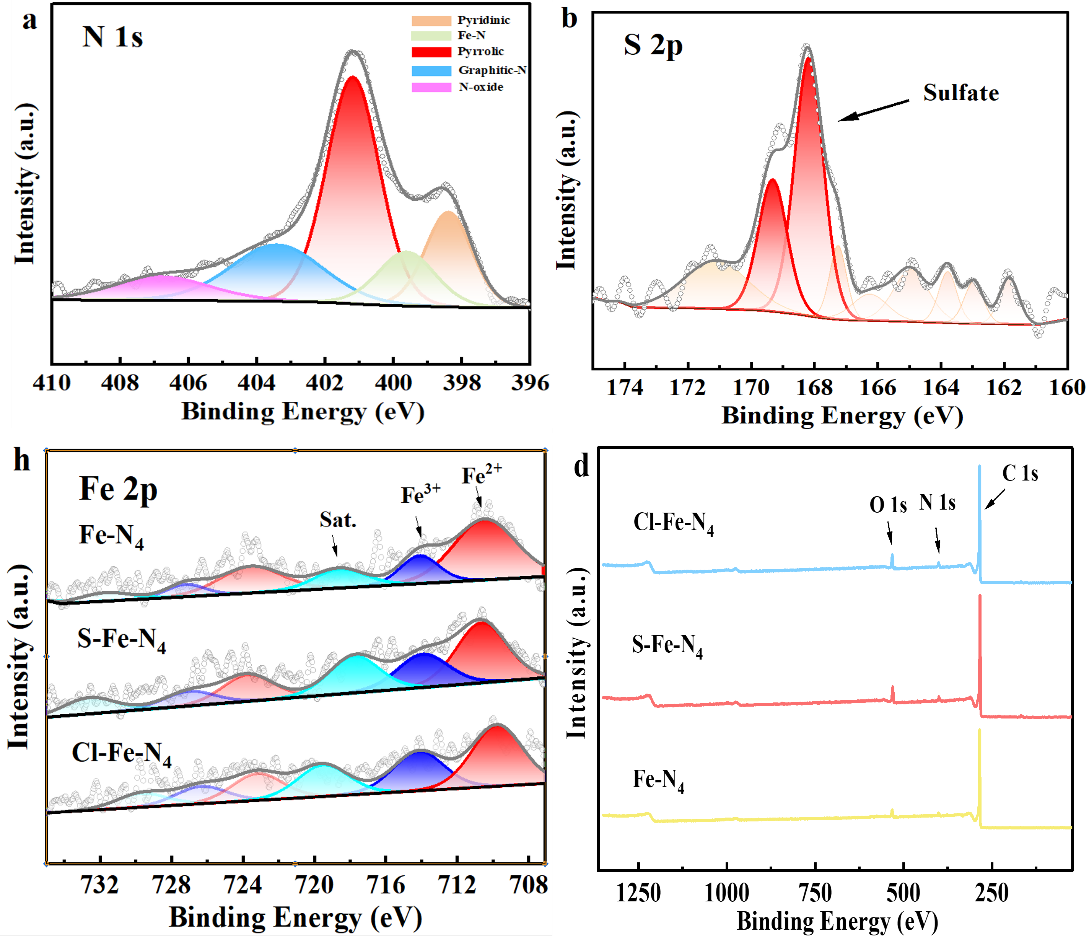


**Fig.** **S4** Deconvoluted high-resolution XPS spectra of **a**) N 1s, **b**) S 2p for Fe-N_4_. **c**) Fe 2p for Cl-Fe-N_4_, S-Fe-N_4_, and Fe-N_4_. **d**) XPS survey spectrum of Cl-Fe-N_4_, S-Fe-N_4_ and Fe-N_4_


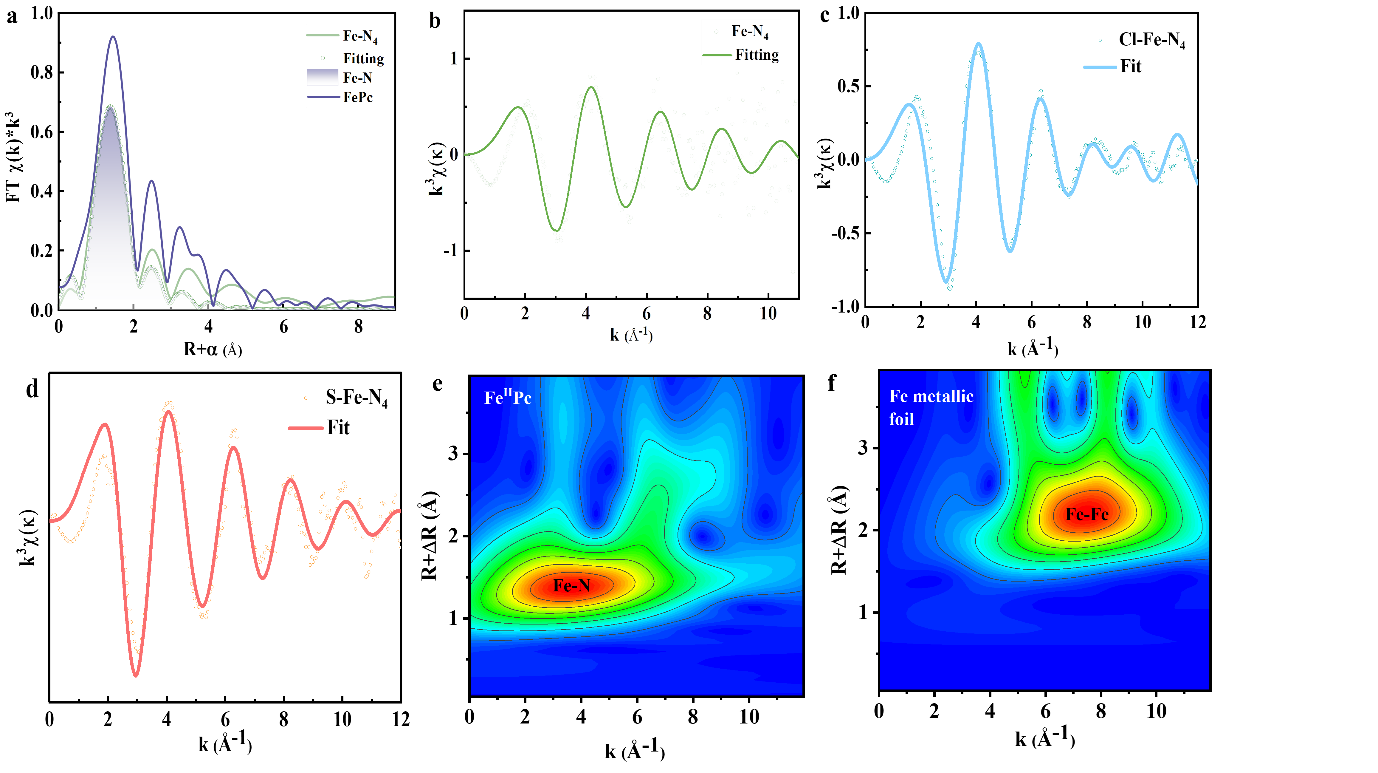


**Fig. S5** k^3^-weighted Fe k-edge EXAFS fitting curves of **a**) Fe-N₄ and reference samples in k space. EXAFS fitting curve of **b**) Fe-N₄, **c**) Cl-Fe-N₄ and **d**) S-Fe-N₄ in R space. Wavelet transform EXAFS analysis of the Fe k-edge for **e**) Fe metallic foil and **f**) FeᴵᴵPc


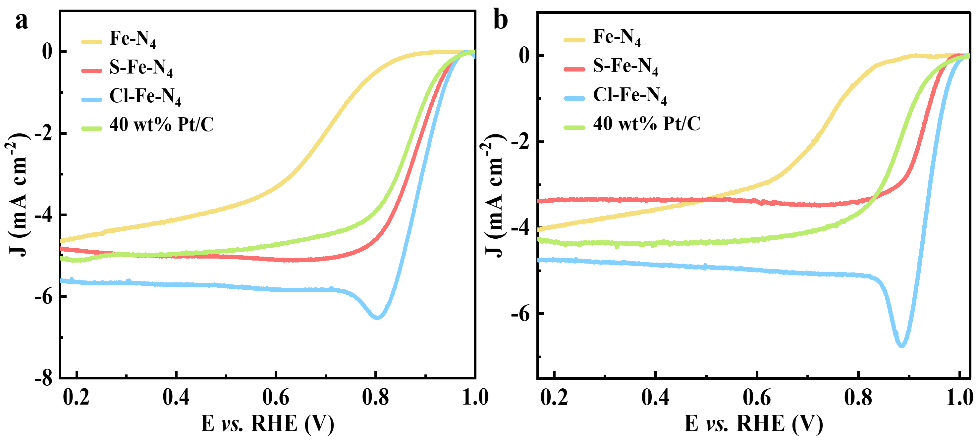


**Fig. S6** LSV polarization curves of Cl-Fe-N_4_, S-Fe-N_4_, Fe-N_4_ and Pt/C using RDE at 1600 rpm in **a**) 0.1 M KOH, **b**) 0.1 M KOH with 0.5 M KCl. Catalyst loading: 0.75 mg cm⁻²


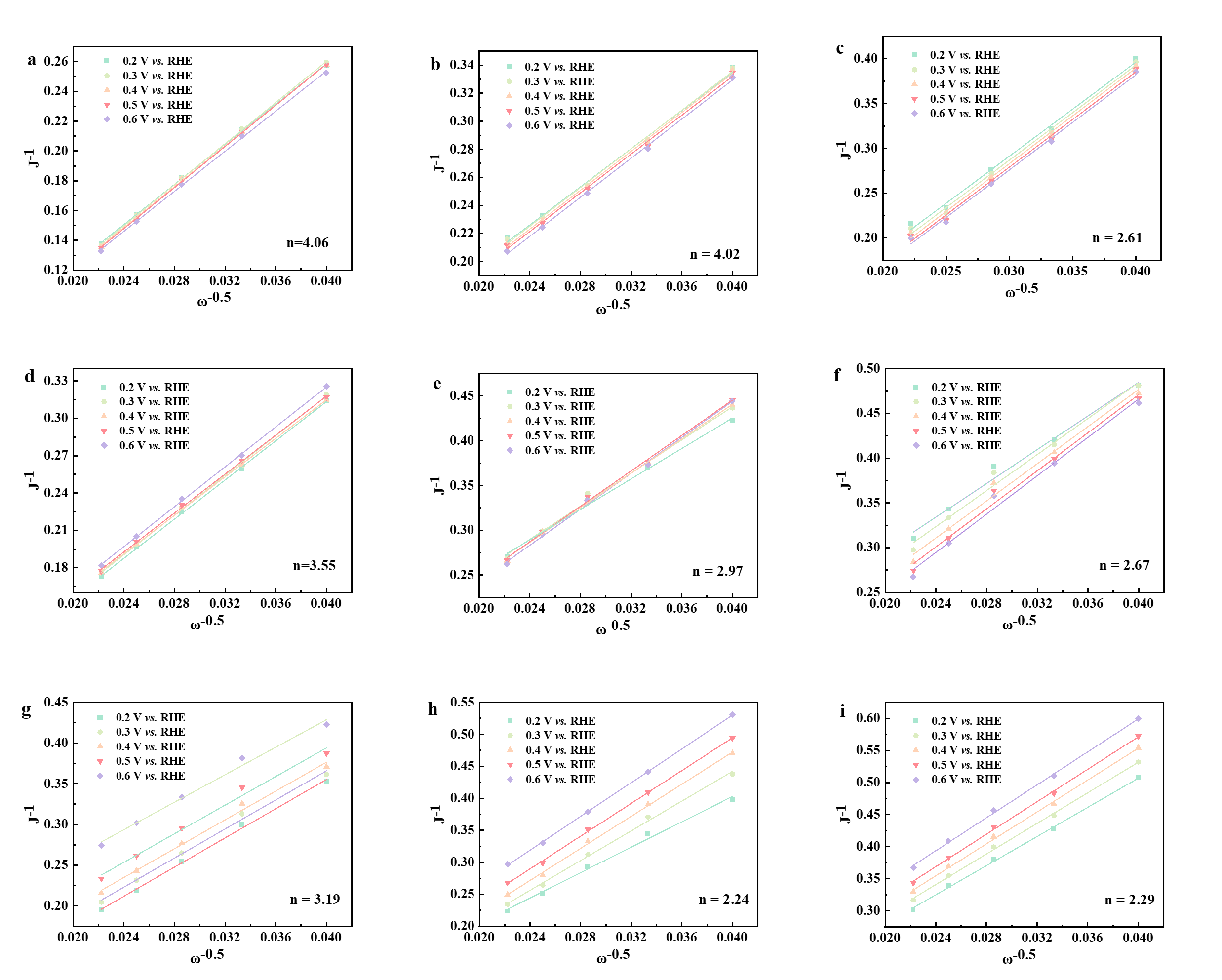


**Fig.** **S7** K-L plots at different rotating speeds in **a**) 0.1 M KOH, **b**) 0.1 M KOH with 0.5 M KCl and **c**) alkaline synthetic seawater (pH = 13) of Cl-Fe-N_4_; in **d**) 0.1 M KOH, **e**) 0.1 M KOH with 0.5 M KCl and **f**) alkaline synthetic seawater of S-Fe-N_4_; **g**) 0.1 M KOH, **h**) 0.1 M KOH with 0.5 M KCl and **i**) alkaline synthetic seawater of Fe-N_4_


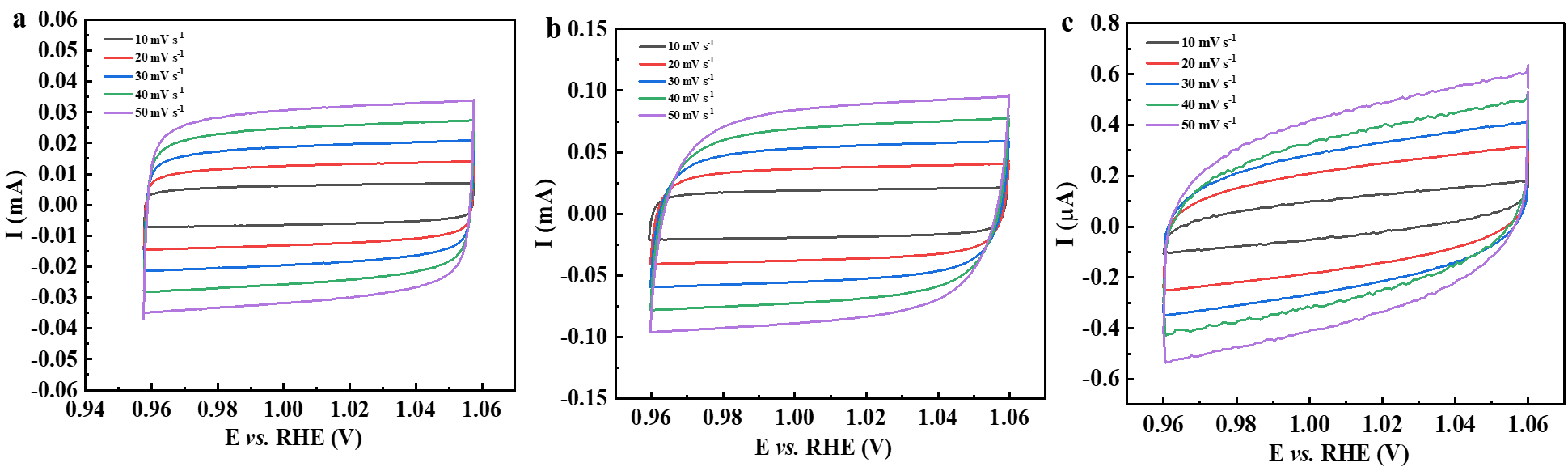


**Fig. S8** CV curves of **a**) Cl-Fe-N_4_, **b**) S-Fe-N_4_, **c**) Fe-N_4_ in alkaline synthetic seawater. Catalyst loading: 0.1 mg cm⁻²


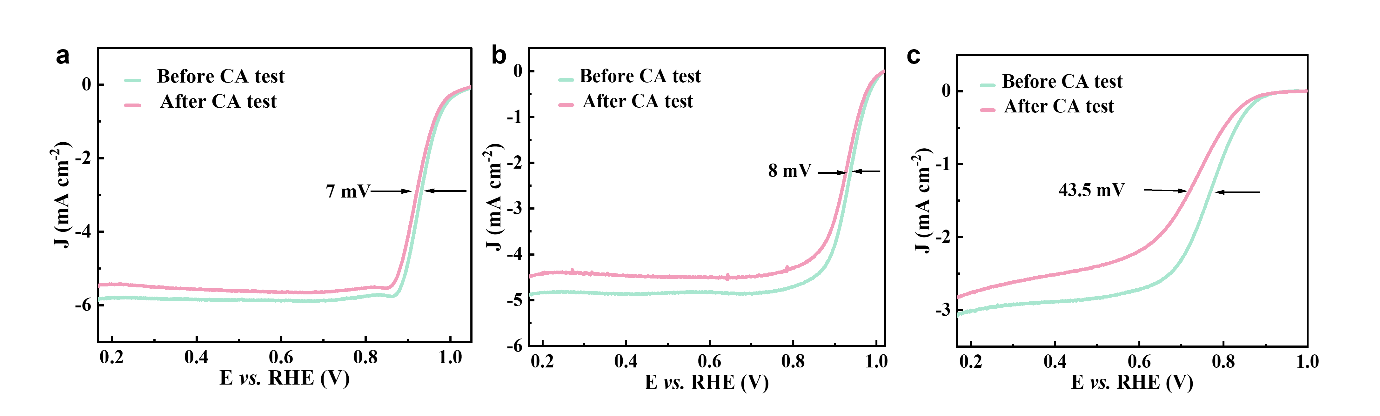


**Fig.** **S9** LSV polarization curves before and after CA test of **a**) Cl-Fe-N_4_, **b**) S-Fe-N_4_, **c**) Fe-N_4_ using RDE at 1600 rpm in alkaline synthetic seawater (pH=13) with a scan rate of 10 mV s⁻¹. Catalyst loading: 0.75 mg cm⁻².


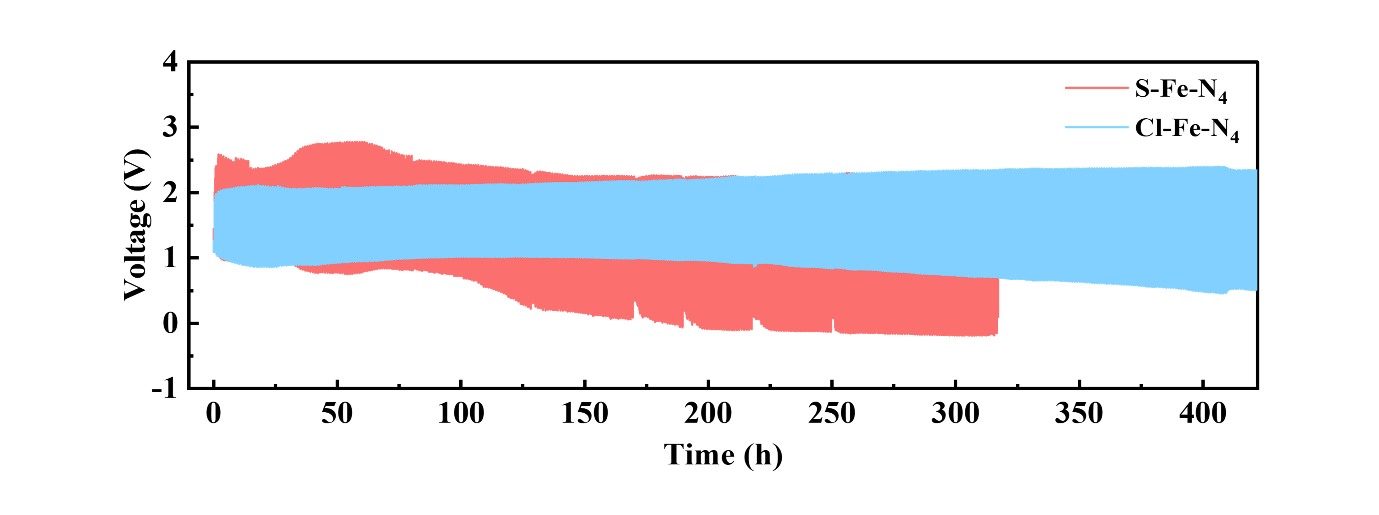


**Fig.** **S10** The long cycling curves of Cl-Fe-N_4_ and S-Fe-N_4_ at 10 mA cm^−2^ in alkaline synthetic seawater

**
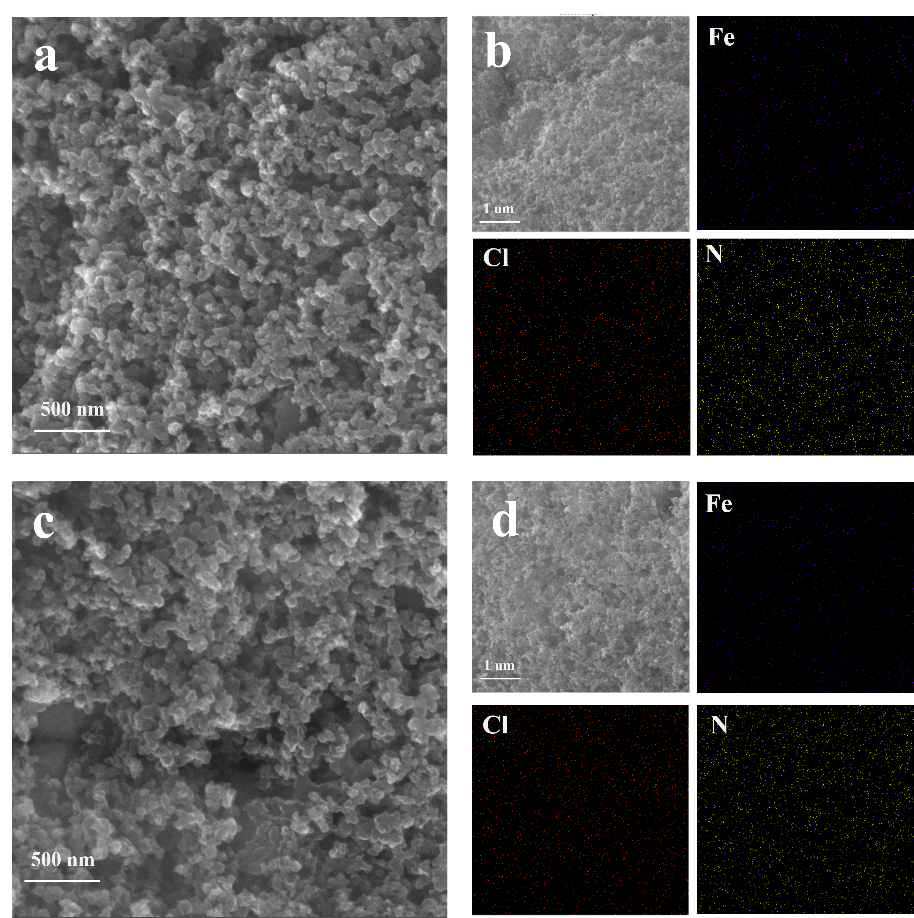
**

**Fig. S11** **a**) SEM pattern and **b**) EDS mapping before the zinc-air battery test, and **c**) SEM pattern and **d**) EDS mapping after the test of Cl-Fe-N_4_


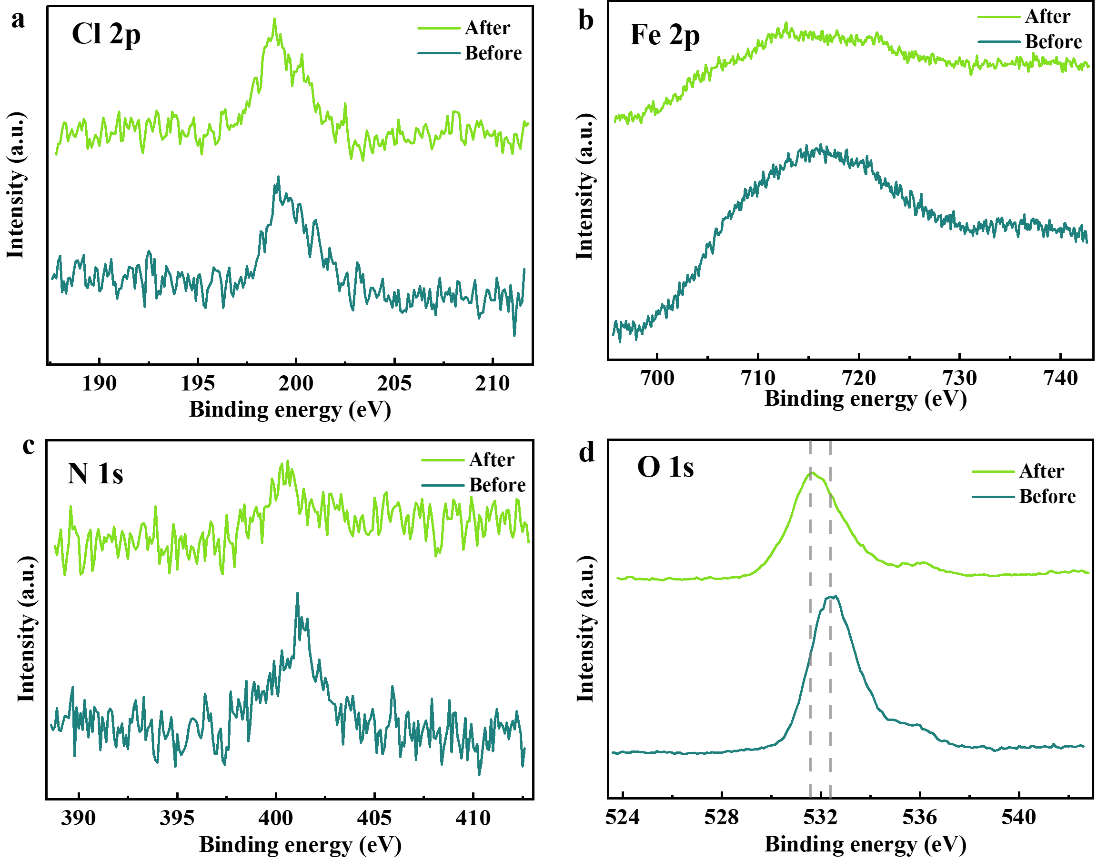


**Fig.** **S12** Deconvoluted high-resolution XPS spectra of **a**) Cl 2p, **b**) Fe 2p, **c**) N 1s and **d**) O 1s for Cl-Fe-N_4_ before and after the zinc-air battery test


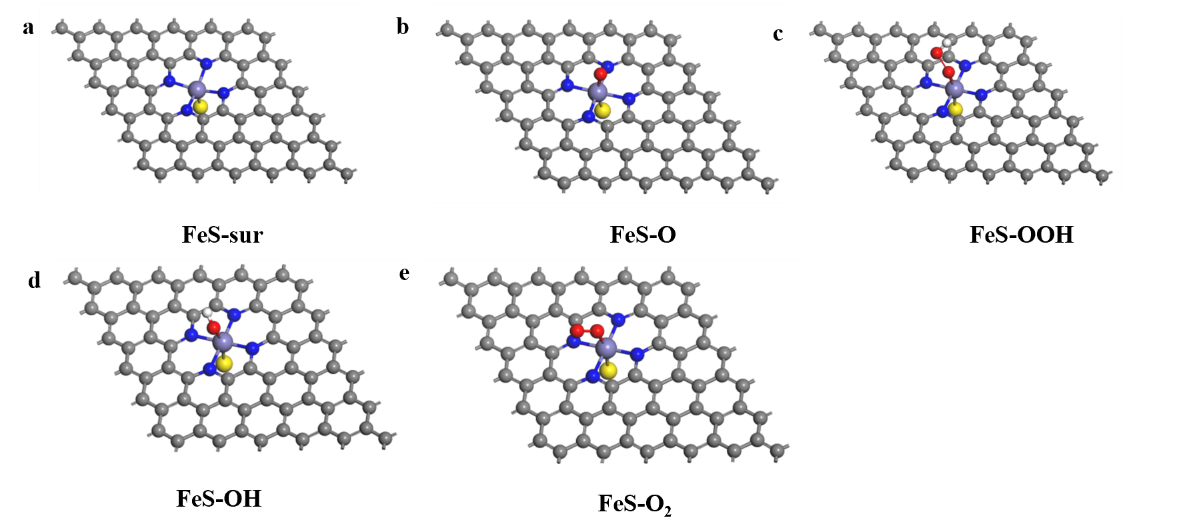


**Fig.** **S13** Theoretical structures of the surface of **a**) unoccupied Fe-N_4_ single atom site, Fe-N_4_ single atom site adsorbed by **b**) -O, **c**) -OOH, **d**) -OH, **e**) -O_2_


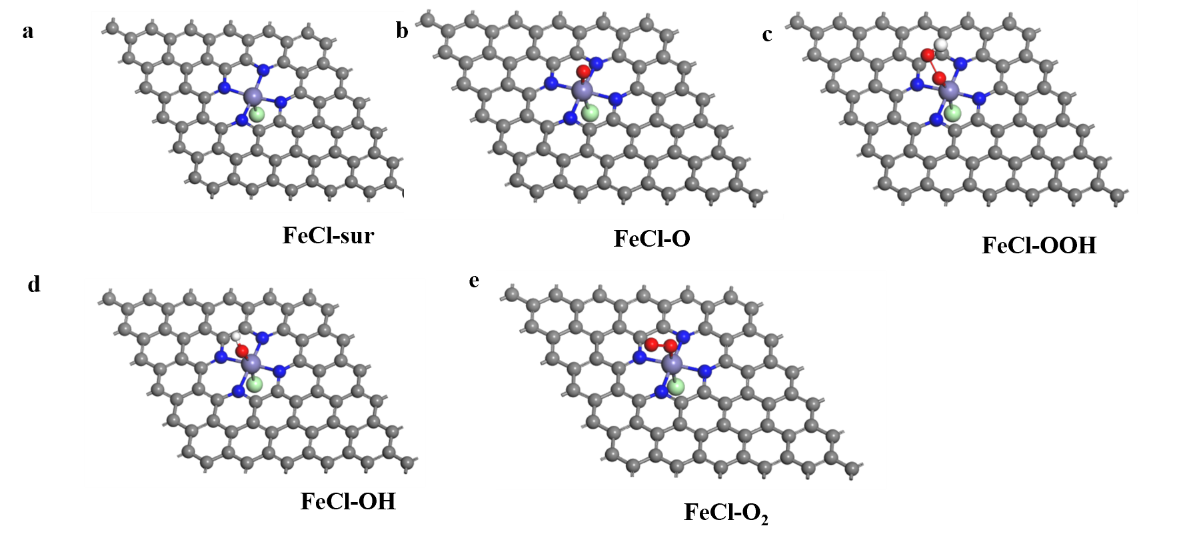


**Fig.** **S14** Theoretical structures of the surface of **a**) unoccupied S-Fe-N_4_ single atom site, S-Fe-N_4_ single atom site adsorbed by **b**) -O, **c**) -OOH, **d**) -OH, **e**) -O_2_


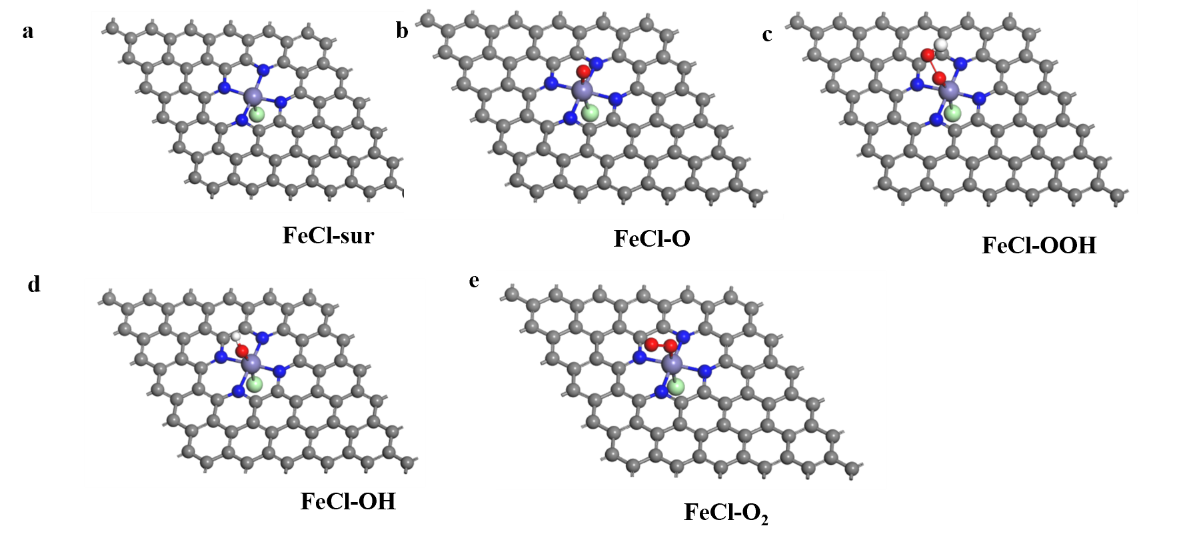


**Fig.** **S15** Theoretical structures of the surface of **a**) unoccupied Cl-Fe-N_4_ single atom site, Cl-Fe-N_4_ single atom site adsorbed by **b**) -O, **c**) -OOH, **d**) -OH, **e**) -O_2_

**
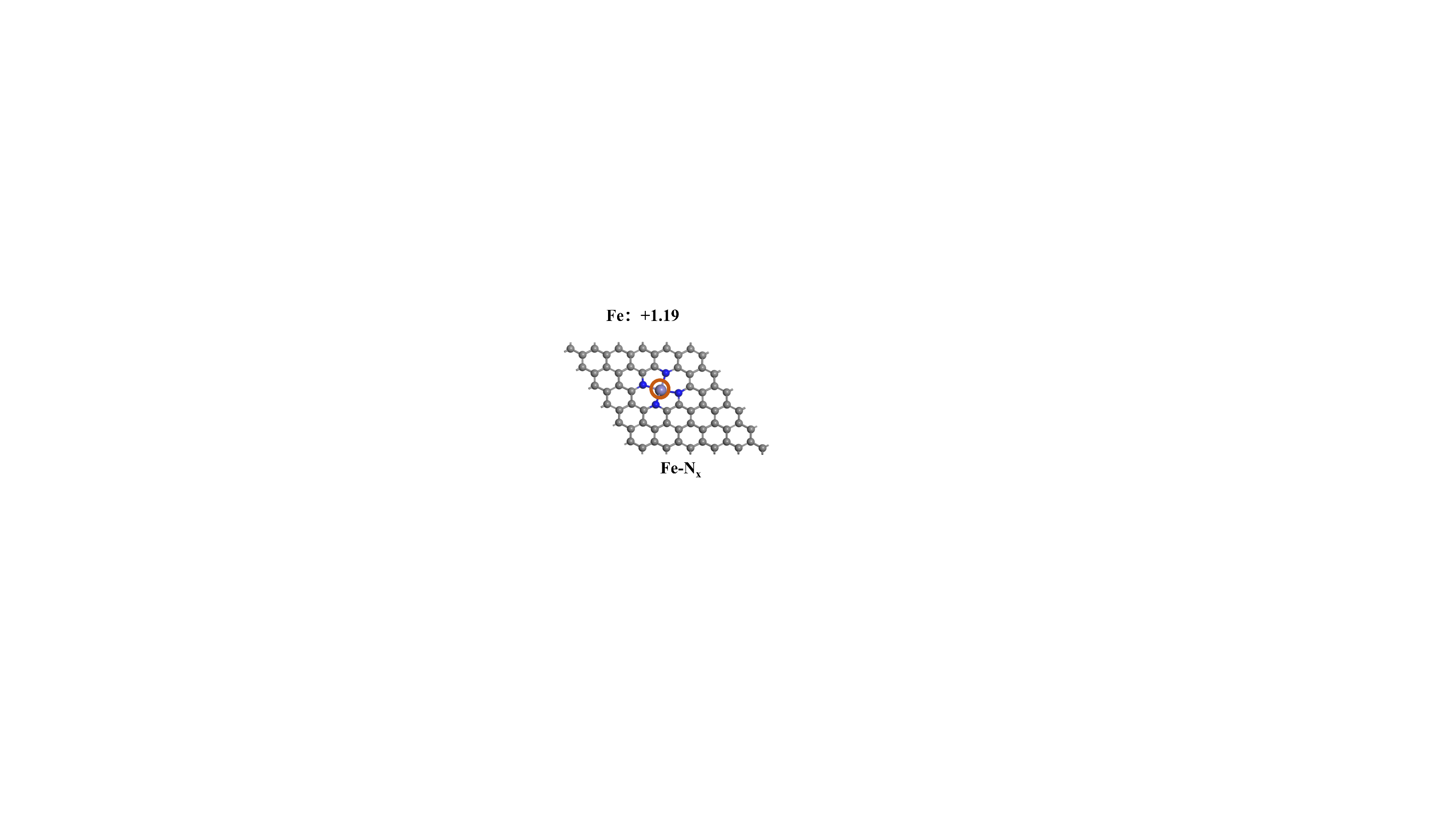
**

**Fig. S16** The Bader charge of Fe-N_4_. Purple ball: Fe; Green ball: Cl; Yellow ball: S; Grey ball: C; Blue ball: N; Red ball: O; White ball: H


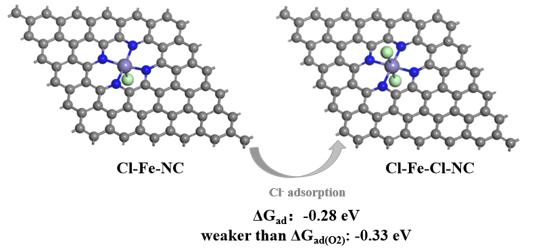


**Fig.** **S17** Adsorption Energy and Configuration of Cl⁻ on Cl-Fe-NC

**Supplementary Tables**

**Table S1** Atomic ratios in different samples based on XPS analysis

|  | C | N | O | S | Cl | Fe |
| --- | --- | --- | --- | --- | --- | --- |
| Cl-Fe-N_4_ | 88.62 % | 4.73 % | 6.00 % | 0.36 % | 0.34 % | 0.13 % |
| S-Fe-N_4_ | 88.21 % | 4.21 % | 6.90 % | 0.81 % | \ | 0.11 % |
| Fe-N_4_ | 92.15 % | 3.55 % | 4.14 % | 0.08 % | \ | 0.10 % |

**Table S2** N species ratios in different samples based on XPS analysis

|  | pyrrolic | pyridinic | Fe-N | N-oxide | graphitic-N |
| --- | --- | --- | --- | --- | --- |
| Cl-Fe-N_4_ | 51.59 % | 23.49 % | 0.34 % | 7.55 % | 17.03 % |
| S-Fe-N_4_ | 53.92 % | 15.72 % | 5.88 % | 7.3 % | 17.18 % |
| Fe-N_4_ | 44.49 % | 16.25 % | 11.54 % | 8.54 % | 19.18 % |

**Table S3** Curve fitting parameters of Fe K-edge EXAFS

| Sample^a^ | Path | N^b^ | R(Å) ^c^ | σ^2^(10^-3^Å^2^) ^d^ | ΔE_0_(eV) | R-factor |
| --- | --- | --- | --- | --- | --- | --- |
| S-Fe-N_4_ | Fe-N | 4.40 | 2.02 | 7.18 | -0.56 | 0.016 |
|  | Fe-S | 0.90 | 2.67 | 30.05 |  |  |
| Cl-Fe-N_4_ | Fe-N | 4.04 | 1.91 | 6.34 | -9.92 | 0.009 |
|  | Fe-Cl | 0.78 | 2.17 | 0.04 |  |  |
| Fe-N_4_ | Fe-N | 4.0 | 1.96 | 5.64 | -4.49 | 0.014 |

^a^S_0_^2^was fixed as 1.0. Data ranges: 3.0 < k ≤ 8.0 Å^-1^,1.0 ≤ R < 3.0 Å.

^b^N is the coordination number.

^c^R is the distance between absorber and backscatter atoms. ^d^σ^2^is the Debye-Waller factor. R-factor is residual factor.

**Table S4** Atomic ratios of Cl-Fe-N_4_ before and after zinc-air battery test based on XPS analysis

|  | C | N | O | Fe | Cl |
| --- | --- | --- | --- | --- | --- |
| Before the test | 75.69% | 4.11% | 19.46% | 0.15% | 0.59% |
| After the test | 67.31% | 3.07% | 28.45% | 0.09% | 1.08% |
